# Pathology exposure in Spanish medical schools: a review of forty-one institutions with insights into pathology curriculum for an international approach

**DOI:** 10.1016/j.acpath.2025.100228

**Published:** 2025-12-01

**Authors:** Francisco Reyes-Albaladejo, Meredith K. Herman, Sara del Río-Ortega, Eduardo Alcaraz-Mateos, Kamran Mirza, Luca Cima

**Affiliations:** a University of Valencia, Valencia, Spain; b University of Michigan Department of Pathology, Ann Arbor, MI, USA; c Universidad de Murcia Department of Pathology, Murcia, Spain; d Department of Diagnostic and Public Health, Section of Pathology, University and Hospital Trust of Verona, Verona, Italy

**Keywords:** Curriculum, Medical training, Pathology education, Residency selection, Spanish medical schools

## Abstract

Pathology plays a vital role in connecting basic science with clinical medicine, yet it remains a lesser-emphasized specialty in Spanish medical education. This study researched all 41 accredited medical schools in Spain to assess how students are exposed to pathology. Findings revealed considerable variability in the presence and structure of pathology education. Only 41.5 % of the institutions had dedicated pathology departments, though nearly all included histology or anatomic pathology with an average instruction span of one academic year each. Public medical schools generally offered more exposure to pathology than private ones. However, elective opportunities focused on pathology were scarce, and only a small proportion of institutions provided international experiences in the United States. These limitations in exposure, elective options, and global collaboration may contribute to the specialty's low ranking among students selecting residency training programs in Spain. The results highlight the need for greater standardization across curricula and suggest that expanding elective courses, integrating digital pathology tools, and fostering international partnerships could significantly enhance student engagement and interest in pursuing pathology as a career.

## Introduction

Pathology plays a crucial role in medical education, contributing to the development of well-rounded physicians with a deep understanding of disease processes. The integration of pathology into the curriculum is vital for equipping students with the knowledge necessary for clinical practice and diagnostic reasoning. In Spain, the medical degree is structured around a six-year curriculum, with the first two years predominantly focusing on basic sciences, such as biochemistry, histology, anatomy, and biostatistics. These early years typically lack significant exposure to clinical practice, which is reserved for the final four clinical years.

The pathology curriculum in Spanish medical schools is designed to provide students with a comprehensive understanding of diseases, including their classification, diagnosis, and pathophysiology. Despite the significant role of pathology in medical education, there remains a notable gap in understanding students’ perceptions of the subject.

**Fig. 1:**
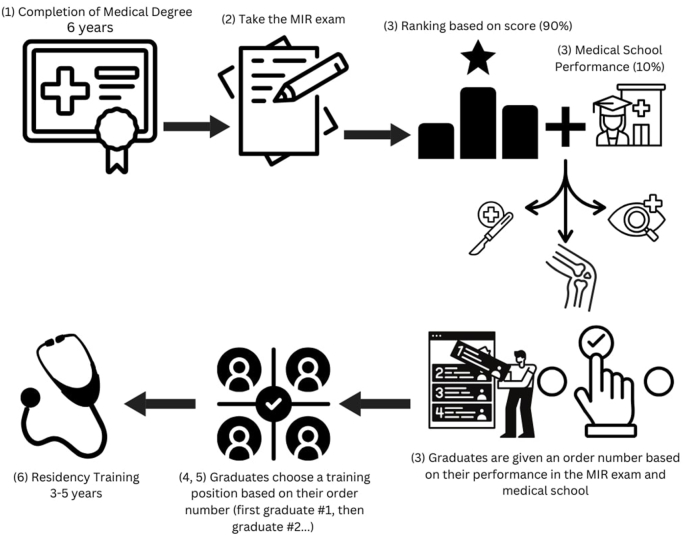
Timeline from medical school to residency program training for a medical student in Spain.

In Spain, the ranking of medical specialties is determined through the *Médico Interno Residente* (MIR) exam,^1^ which medical graduates must pass to specialize. The pathway to becoming a medical specialist involves completing a six-year medical degree (1), followed by the annual MIR exam (2). This exam tests medical knowledge across various fields, with 90 % of the final score based on MIR performance and 10 % on medical school performance. The MIR exam has items for every discipline, including pathology; there is no data suggesting that the distribution of questions per discipline influences career choices among test-takers (3). Candidates’ rankings determine their order number for selecting a specialty from available positions (4). The matching process allocates residency spots, with higher-ranked candidates selecting first (5). Once a candidate secures a spot, they undergo a 3- to 5-year residency program, depending on the specialty (6) (Fig. 1).

In contrast to the National Resident Matching Program matching process, where letters of recommendation, clinical experience, interviews, and other factors are holistically reviewed and influence the graduates’ match to specific training programs, Spain’s system operates differently. In Spain, institutions do not select graduates for programs. Instead, graduates choose their desired training position, specialty, and location, regardless of previous experience. The sole determining factor is the candidate’s performance on the MIR exam, with a minor influence from medical school performance.

According to the 2023–2024 MIR^1^ exam results, pathology is one of the least chosen specialties, ranking 34th out of 45 in terms of preference among graduating medical students, with 81 anatomic pathology programs offered and a total of 132 positions. The dynamics of how residency positions are chosen allows us to extrapolate the interest for a specialty based on the order list/rank median by which it is chosen. A lower median means that the more competitive graduates who scored higher (and choose earlier in the order list) and have more freedom to choose a program are choosing that specialty to train as residents. Whereas, a specialty with higher median means that lots of graduates who have decided on their residency earlier have not chosen that specialty, thus it is not as attractive to high-scoring graduates.^1^

Based on the survey data from Martínez-Ciarpaglini et al., the satisfaction from the training in Spain is due to two main aspects: previous knowledge of the specialty and the quality of the training. While high-quality training is understandably linked to greater satisfaction, it is concerning that prior exposure—typically gained during medical school—is such a strong predictor.^2^ This raises the questions: is how pathology is exposed to students in medical schools’ curricula a factor contributing to the comparatively low appeal of pathology? Does the exposure affect the satisfaction from the training received after choosing pathology?

To address this, a study exploring how students are exposed to pathology in Spanish medical schools and its impact on students’ perceptions and career choices is crucial. Nevertheless, it must be addressed that a holistic approach to this matter, including compensation, post-residency workforce accessibility, and other factors also contribute to the current comparatively low appeal of pathology.

Unlike the United States (US) pathology residency training, where there is a potential combination of anatomic pathology/clinical pathology or training of one of the two modules; in Spain, there is only anatomic pathology training. However, within the expected skills of an anatomic pathology resident, some clinical pathology aspects are included. The remaining roles performed by clinical pathologists are covered by doctors in other specialties (clinical biochemistry, hematology, etc.) and other laboratory professionals.^3^

Medical schools in Spain, both public and private, have the autonomy to design their official academic programs within the mandatory framework provided by the government, adhering to European agreements.^4^ Once a program is developed, it must undergo two critical steps for approval: the VERIFICA process, managed by the Council of Universities, and authorization by the corresponding autonomous community. For the VERIFICA process, a favorable evaluation by a competent agency is required before the Council of Universities can proceed. These steps, although beneficial for securing homogeneity in medical education, may hinder the progress to adapt for medical school needs or interests.^5^

Spanish teaching model, through the previously mentioned framework,^5^ emphasizes an integrated approach through 4-to-5-month terms where different subjects are taught simultaneously both at a theoretical (lectures and seminar) and a practical level (laboratory visits, daily rotations at one hospital service related to one of the subjects…); and command of each subject is typically assessed by a final multiple choice question exam. Unlike the US, which emphasizes integration with variable-length rotations at hospital services where the basis or theoretical knowledge has been previously taught, rather than being taught at the same time as the rotations.

In this study, we explore the available data regarding histology and anatomic pathology training during medical school years, as well as potential electives and international exposure offered through Spanish medical schools in the hopes of understanding what is the current situation and what can be done to improve national and international interest in pathology and decrease dropout rates in Spain. We aim to update and expand on a subject that was previously explored by Leiva-Cepas^6^ and Tarragona et al.^7^ by including private medical schools in Spain as well as by including updated data on the topic; by including private medical schools, we broaden the picture of how anatomic pathology is taught in Spain.

## Materials and methods

A thorough manual review of publicly available online information from accredited public and private medical schools in Spain was carried out over a six-week period from August 20, 2024, to October 1, 2024. The review aimed to collect data on key aspects of medical education, including program location, type (public or private), language of instruction, class size, and duration. Additionally, the authors analyzed the structure of medical curricula, focusing on the length of study, the organization of preclinical and clinical education, and the integration of histology and anatomic pathology courses. The reason of including histology and no other subjects such as anatomy is that the subject of histology during medical school is heavily linked to anatomic pathology in terms of continuity amongst topics included and the way it is taught since the faculty responsible for teaching histology is usually part of the Pathology Department; unlike other subjects such as anatomy or biochemistry, despite having undeniable importance for pathology practice.

A thorough analysis of course syllabi, required and elective coursework, and clinical rotations was conducted to determine the extent of exposure medical students receive to histology and anatomic pathology. To assess this exposure, data on histology and anatomic pathology education provided by each institution were collected, including detailed descriptions of courses, lecture hours, laboratory sessions, clinical exposure, key learning objectives, expected competencies, and evaluation methods. The presence of histology and anatomic pathology departments within each medical school was also investigated, along with information on faculty size, including the number of full-time and part-time staff, and the student-to-faculty ratio. Data on agreements or partnerships with US institutions that allow for elective rotations were identified.

### Data analysis

The collected data were summarized to provide an overview of the general medical education landscape in Spain, with a focus on pathology education. Descriptive statistics were applied to quantify differences in pathology exposure across institutions.

### Data validation and reliability

Throughout the data collection and analysis process, steps were taken to ensure the reliability and validity of the collected information. Data sourced from university websites and publications were cross-verified with other available resources and, where possible, confirmed via direct communication with university representatives. Consistency checks were performed to ensure accurate and uniform categorization and summarization.

Efforts were made to reduce potential bias by standardizing the criteria for data inclusion and having multiple authors independently review and confirm the collected data. These measures ensured the accuracy and reliability of the findings, providing a comprehensive understanding of the role of pathology in medical education in Spain.

## Results

A total of forty-one (N = 41) Spanish medical schools were identified in the search and all medical schools were included in the analysis (Table 1^8^, ^9^, ^10^, ^11^, ^12^, ^13^, ^14^, ^15^, ^16^, ^17^, ^18^, ^19^, ^20^, ^21^, ^22^, ^23^, ^24^, ^25^, ^26^, ^27^, ^28^, ^29^, ^30^, ^31^, ^32^, ^33^, ^34^, ^35^, ^36^, ^37^, ^38^, ^39^, ^40^, ^41^, ^42^, ^43^, ^44^, ^45^, ^46^, ^47^, ^48^). Ten medical schools were listed as private institutions (N = 10, 24.3 %) while 31 were listed as public institutions (n = 31, 75.6 %). The average class size was 177 students, ranging from 50 to 385 students (private institution range: 50–200; public institution range: 60–385). Seventeen institutions had a pathology department (N = 17, 41.5 %) and twenty-four had no pathology department (n = 24, 58.5 %). The data on quantity of pathology faculty was only available at 9 public institutions and 1 private institution, with an average size of 29 faculty (range: 1 to 66 pathology faculty). In the remaining schools, manual research showed that experts in biomedical sciences and anatomic pathology specialists taught histology and anatomic pathology subjects.

**Table 1:** Overview of Spanish medical schools: class size, pathology/histology focus, electives, and rotation opportunities.

Medical School	Type	Location	Class size (year)	Pathology/histology-focused subjects	Time of exposure (academic years)	Time in histology (years)	Time in pathology (years)	Pathology/histology-focused electives	Pathology Department	Pathology faculty size	USA elective/rotation opportunities	English group
Universidad Alfonso X el Sabio^8^	Private	Madrid	180	Yes	1.5	0.5	1	No	No	NA^a^	No	No
Universidad Autónoma de Barcelona^9^	Public	Barcelona	385	Yes	2.5	1.5	1	No	Yes	NA^a^	No	No
Universidad Autónoma de Madrid^10^	Public	Madrid	260	Yes	4	1	3	Yes	Yes	22	No	No
Universidad Católica de Valencia San Vicente Mártir^11^	Private	Valencia	120	Yes	0.5	0	0.5	No	No	NA^a^	Yes	No
Universidad Católica San Antonio^12^	Private	Murcia	90	Yes	1	0.5	0.5	No	No	NA^a^	No	No
Universidad CEU Cardenal Herrera^13^	Private	Castellón	50	Yes	2	1	1	No	No	NA^a^	No	Yes
Universidad Complutense de Madrid^14^	Public	Madrid	320	Yes	2	1	1	Yes	Yes	NA^a^	No	Yes
Universidad de Alcalá^15^	Public	Alcalá	60	Yes	3	2	1	No	No	NA^a^	Yes	No
Universidad de Barcelona^16^	Public	Barcelona	300	Yes	2	1	1	No	Yes	66	No	Yes
Universidad de Cádiz^17^	Public	Cádiz	180	Yes	4	2	2	No	Yes	34	No	No
Universidad de Cantabria^18^	Public	Cantabria	124	Yes	2	1	1	No	No	NA^a^	No	No
Universidad de Castilla La Mancha^19^	Public	Castilla La Mancha	216	Yes	2	1	1	No	No	NA^a^	No	No
Universidad de Córdoba^20^	Public	Córdoba	120	Yes	3	2	1	No	Yes	19	No	No
Universidad de Extremadura^21^	Public	Extremadura	132	Yes	1	0.5	0.5	No	Yes	1	No	No
Universidad de Girona^22^	Public	Girona	92	No	NA	NA	NA	No	No	NA^a^	No	No
Universidad de Granada^23^	Public	Granada	272	Yes	2	1	1	No	Yes	19	Yes	No
Universidad de La Laguna^24^	Public	San Cristóbal de La Laguna	109	Yes	2	1	1	No	No	NA^a^	No	No
Universidad de Las Palmas de Gran Canaria^25^	Public	Las Palmas de Gran Canaria	150	Yes	2	1	1	Yes	No	NA^a^	No	No
Universidad de Les Illes Balears^26^	Public	Islas Baleares	69	Yes	1.5	1	0.5	No	No	NA^a^	No	Yes
Universidad de Lleida^27^	Public	Lleida	132	Yes	2	1	1	No	No	NA^a^	No	Yes
Universidad de Málaga^28^	Public	Málaga	170	Yes	4	2	2	No	Yes	NA^a^	NA^a^	Yes
Universidad de Murcia^29^	Public	Murcia	220	Yes	3	1	2	No	No	NA^a^	No	No
Universidad de Navarra^30^	Private	Navarra	200	No	NA	NA	NA	No	Yes	42	No	Yes
Universidad de Oviedo^31^	Public	Oviedo	165	Yes	2	1	1	Yes	No	NA^a^	No	Yes
Universidad de Salamanca^32^	Public	Salamanca	207	Yes	1.5	1	0.5	No	Yes	36	No	No
Universidad de Santiago de Compostela^33^	Public	Santiago de Compostela	360	Yes	4	2	2	No	No	NA^a^	No	No
Universidad de Sevilla^34^	Public	Sevilla	352	Yes	1.5	0.5	1	No	Yes	NA^a^	No	No
Universidad de Valencia^35^	Public	Valencia	336	Yes	2	1	1	No	Yes	36	No	Yes
Universidad de Valladolid^36^	Public	Valladolid	219	Yes	1	0.5	0.5	No	Yes	NA^a^	Yes	No
Universidad de Vic^37^	Private	Vic	115	Yes	NA	NA	NA	No	No	NA^a^	No	No^b^ (some electives are taught in English)
Universidad de Zaragoza^38^	Public	Zaragoza	220	Yes	1.5	1	0.5	No	Yes	NA^a^	Yes	No
Universidad del País Vasco^39^	Public	País Vasco	200	Yes	2	1	1	No	No	NA^a^	No	No^☨^ (some subjects are taught in English)
Universidad Europea de Madrid^40^	Private	Madrid	200	Yes	1.5	0.5	1	No	No	NA^a^	No	No
Universidad Francisco de Vitoria^41^	Private	Madrid	140	Yes	2	1	1	No	No	NA^a^	No	No
Universidad Internacional de Cataluña^42^	Private	Cataluña	100	Yes	0.5	0	0.5	No	No	NA^a^	Yes	No^b^ (some subjects are taught in English)
Universidad Jaume I^43^	Public	Castellón	85	Yes	1	0	1	No	Yes	NA^a^	No	No
Universidad Miguel Hernández^44^	Public	Elche	130	Yes	1.5	0.5	1	No	Yes	23	No	No
Universidad Pompeu Fabra^45^	Public	Barcelona	60	Yes	4	3	1	No	No	NA^a^	Yes	No^b^ (some subjects are taught in English)
Universidad Rey Juan Carlos^46^	Public	Madrid	158	Yes	2	1	1	No	No	NA^a^	No	No
Universidad Rovira I Virgili^47^	Public	Tarragona	130	Yes	3	2	1	No	No	NA^a^	No	Yes
Universidad San Pablo-CEU^48^	Private	Madrid	160	Yes	3	2	1	Yes	No	NA^a^	No	No

a Data not available, not applicable (e.g., no pathology department) or insufficient for accurate analysis (e.g., temporary teachers on staff, professors who teach different subjects, research-only personnel …).

b Majority of subjects are taught in Spanish, with a minor subset being taught in English.

The majority of medical schools had pathology- and histology-focused subjects in their curriculum (N = 39, 95.1 %) while two programs did not, which means that they included the basic topics of these disciplines within other subjects being taught. The average time spent with at least one subject covering extensive histology or anatomic pathology contents in academic years was 2.13 years, ranging from 0.5 (half a year, semester) year to 4 years (four academic years). The average duration of dedicated histology instruction was 1.07 academic years, with a range spanning from 0 to 2 years (SD: 0.65). Similarly, the average duration of dedicated pathology instruction was 1.05 years, with a range extending from 0.5 to 3 years (SD: 0.52). Public schools had significantly more total exposure and pathology years than private schools, suggesting that they might have the resources through public funding to have on-site anatomic pathology laboratories and personnel. Despite having no data available on correlation between increased time exposed to histology/anatomic pathology and residency choices, it was clear from Martínez-Ciarpaglini et al.^2^ that residency satisfaction was linked to prior knowledge of the discipline; further research on whether knowledge of the discipline is characterized by more qualitative and/or quantitative exposure is unknown. However, the difference in histology years was not statistically significant (*P* = 0.0805), suggesting no strong difference between public and private institutions in this area (Table 2).

**Table 2:** Paired *t*-test comparing histology and pathology exposure between public and private Spanish medical schools. The units (except for *P*-value) are in *academic* years, not *calendar* years.

Category	Public mean (years)	Public SD (years)	Private mean (years)	Private SD (years)	*t*-test *P*-value
Total Exposure	2.30	0.94	1.50	0.85	0.0386
Histology Years	1.18	0.62	0.69	0.65	0.0805
Pathology Years	1.12	0.55	0.81	0.26	0.0345

Only five programs had specific electives focused on pathology and histology, four of which were public institutions, whereas the majority of programs had none (N = 35, 85.3 %). Pathology elective rotations in the US were available through affiliated programs at only seven institutions (n = 7, 17.07 %) (Table 3^11^^,^^15^^,^^23^^,^^36^^,^^38^^,^^42^^,^^45^). No research has been done on the reasons behind this lack of international collaborations between Spain and the US, though—as per the authors’ own experience—rather than lack of interest by the alumni in American rotations, it seems that agreements by different educational and medical systems (largely public in Spain and largely private in the US) are a challenge in terms of funding and institutional support. Additionally, 10 institutions offered a group or class for students who wished to learn medicine in English (N = 10, 24.3 %); Four different institutions only offered some subjects taught in English (N = 4, 9.8 %). There were no available data regarding the number of students and graduates who undergo residency applications to the US. However, it can be inferred that the number of applicants in general and in anatomic pathology in particular is low considering that 15 out of the 41 medical schools in Spain (N = 15, 36.6 %) are currently eligible for 2025 Pathways.^49^

**Table 3:** Spanish medical schools with established agreements for educational experiences in the USA for students.

Medical school	Program	Affiliated Institutions
Universidad Católica de Valencia San Vicente Mártir^11^	Mundus - Prácticas	1. George Washington University 2. West Virginia University
Universidad de Alcalá^15^	Institutional agreement	1. Mississippi State University 2. College of Charleston 3. Wisconsin La Crosse
Universidad de Granada^23^	Institutional (bilateral) agreement	Any (student must be privately accepted by the institution)
Universidad de Valladolid^36^	Institutional agreement	1. University of North Carolina 2. San Diego State University 3. Saint Martin’s University 4. Moravian University
Universidad de Zaragoza^38^	Institutional agreement	1. George Mason U 2. University of Oklahoma 3. Northern Arizona U 4. San Francisco State University 5. The City Uof New York 6. New Jersey City U
Universidad Internacional de Cataluña^42^	Institutional (bilateral) agreement	Any (student must be privately accepted by the institution)
Universidad Pompeu Fabra^45^	Institutional (bilateral) agreement	Reed College

## Discussion

The data presented reveal important insights into the state of pathology education in Spanish medical schools, highlighting both strengths and areas for improvement.

### Prevalence of pathology in the curriculum

The majority of Spanish medical schools (95.1 %) include pathology and histology in their curricula, which demonstrate the foundational role of pathophysiology subjects in medical education. However, there is notable variation in the duration and depth of instruction. On average, students receive around 2 years of education in these subjects but this can range from as little as one semester to up to four years, highlighting inconsistencies in students’ exposure to pathology into the curriculum.

According to the data obtained from our research, students are typically introduced to pathology in the third year through an integrated approach that assumes knowledge in basic science areas such as anatomy, biochemistry, and pharmacology. This integration helps bridge the gap between normal and abnormal physiological processes, laying the foundation for later clinical understanding. The curricula almost invariably include theoretical courses on topics such as general pathology, tumor pathology, and infectious diseases, as well as practical experiences, such as autopsy observations, microscopy sessions, and hands-on laboratory training in histology and molecular pathology, amongst other topics and practical experiences as these are items commonly required for a curriculum in this subject to be approved through the VERIFICA process.^5^

### Electives and specialization opportunities

Few medical schools (12.2 %) offer electives focused specifically on pathology or histology. Given the increasing recognition for specialized expertise in pathology, increasing the availability of elective opportunities could provide students with deeper insights into the field and possibly boost interest in pathology as a specialty.

During medical school, one of the authors explored the possibility of final year rotations in pathology, which did not appear to be listed as an option on the medical school website. This inquiry raised questions about the feasibility of including such rotations. It was noted that incorporating anatomic pathology rotations into the final year curriculum would likely require modifications to the medical school’s VERIFICA, a process known to be administratively complex.^5^ This underscores the challenges that may arise when adapting curriculum to accommodate specific learning opportunities, such as the ones related to anatomic pathology. Thus, it seems that medical schools can easily disregard anatomic pathology rotations without affecting their quality accreditation.

### Clinical and international exposure

A small proportion of medical schools (17.07 %) offer elective rotations or similar experiences in the US, which may limit students’ exposure to different models of pathology practice, clinical environments, and networking ultimately hampering chances of applying for pathology training positions in countries where experience on the specialty (such as the US Match process) matter. Additionally, despite the increasing importance of international learning opportunities, only 24.3 % of institutions offer English language programs for non-Spanish-speaking students, which can hinder access to education for international students or reduce Spain’s visibility as an educational destination for global talent as English is the expected shared language for global communication. It must be noted that students can have international anatomic pathology experience in other countries, prominently European, integrated as part of the curriculum of an exchange program but international experiences focused only on anatomic pathology are not offered.

### Pathology departments and faculty

The presence of dedicated pathology departments in just 41.5 % of institutions may reflect a lack of specialized resources in many Spanish medical schools. Furthermore, the variability in the number of faculty members teaching pathology-related subjects suggests a lack of standardization in faculty expertise and departmental infrastructure since not only pathologists may have the responsibility to teach this discipline. Moreover, there are no specialty boards for certification after residency training; although additional credentials may increase prestige and pay, there are no data implying a significant impact on students’ perception of pathology.

### Pathology’s role in career choices

The relatively low number of students pursuing pathology as a specialty as reflected in the 2023–2024 MIR results could be attributed to several factors. A lack of hospital pathology service exposure, limited elective opportunities, and the variable duration of pathology education might all contribute to students’ perceptions of pathology as less attractive compared to other specialties. Further exploration into students’ attitudes and experiences related to pathology could help identify specific barriers to pursuing this field.

### Training and specialization

The number of new specialists in clinical laboratory medicine, including pathology, has been declining, with a decrease of 7 % annually between 2015 and 2019. The specialties related to clinical laboratory medicine are among the specialties with the lowest demand. The dropout rate was 12–14 %. These percentages indicated that these specialties were not attractive to new physicians, which correlated with the most recent data provided by the residency MIR match process. The reasons why anatomic pathology and, by extent, the rest of clinical laboratory practice is less attractive expand beyond medical school since government-based decisions also suggest less implication on the progress of clinical laboratory specialties: despite the estimated 66 % of clinical decisions made based on laboratory results, only 3 % of health expenditure in Spain is allocated to laboratory tests for the diagnosis and management of patients. A higher percentage of health expenditure should be allocated in laboratory progress to be congruent with its prominence in clinical decisions.^50^

The issue extends beyond the expenditure allocated to laboratory testing; in recent years, pathologists in Spain have echoed their concerns about a shortage of pathologists that may affect the speed and accuracy of disease diagnoses, including cancer.^51^

Despite its importance, pathology seemingly goes unnoticed by medical schools and students in other countries as well. Only 1–3 % of students in Canada and the US go into pathology after graduation,^52^^,^^53^ similar proportions have been seen in Australia and the United Kingdom.^53^ The shortage of pathologists has been deemed severe and long-standing by the Canadian Association of Pathologists. In 2008, a public inquiry into significant laboratory failures in eastern Canada urged Canadian medical schools to “make the specialty of pathology more appealing to prospective candidates.”^54^

A survey of Canadian and international medical residents in Canada revealed that only 7.5 % of non-pathology residents had considered pathology as a residency option. Those in diagnostic radiology, medical genetics, and ophthalmology were more likely to have considered it. Pathology residents cited the field’s attractive nature and good lifestyle as key reasons for their choice, while 75 % of non-pathology residents preferred specialties with direct patient contact, and 18 % reported insufficient exposure to pathology during medical school.^55^^,^^56^ Assuming similar numbers for recent graduates in Spain who do not mind not having patient contact (25 %), and considering that out of all the 2023 MIR applicants who could choose pathology (spots still available) only 1.75 % chose it, we could extrapolate that out of the pool of potentially interested applicants, only 7 % end up choosing pathology (1.75/25).^1^ This percentage is similar to the one obtained by the Canadian survey, where residency application follows a different system. Despite these differences, this further solidifies the notion of the limited appeal of pathology amongst countries.^55^

Medical students commonly perceive pathology as clinically invisible as reported by Hung et al. with variable exposure during medical school and persistent misconceptions about the field. These include stereotypes of pathologists as introverted or antisocial and a view that pathologists are “lesser” or “non-clinical” doctors despite some considering them the “doctor’s doctor.” This lack of awareness and visibility hinders interest in pathology as a career choice. No data have been reported on whether this vision of pathologists remains similar after entering the workforce or it is changed based on variable points of contact with pathologists at work (tumor boards, blood banks, etc.). Generally, medical students in Spain do not experience moments when pathologists interact in person with other physicians nor spend quantitative enough time in a pathology service to understand the relevance of pathology in patient care, hence why all these factors combined explain their perceptions on the field.^53^

Surveys show that pathologists rank highly in job satisfaction (58 %) and fair compensation (63 %) compared to those of other specialties; appreciating career flexibility, intellectual challenges, and recognition as pivotal contributors to patient care.^57^ European pathology trainees similarly valued the field’s quality of life and scientific excitement, though they noted limited cooperation with clinicians and surgeons (not specified in which aspect), inadequate public awareness, and a need for better training and working conditions. Despite these challenges, 84 % of trainees were satisfied with their career choice, viewing pathology as a fascinating and essential specialty with a promising future.^58^

As mentioned, several studies have investigated medical students’ perceptions of pathology in other countries, such as the US, Canada, and Australia. However, these studies may not be generalizable to the Spanish context as medical education systems and cultural factors can influence students’ perceptions and attitudes toward pathology. Nevertheless, as per the author’s own experience in the Spanish medical education system, as well as the data provided by the residency match outcomes in Spain through the MIR process, we believe the data are highly suggestive to be like Spain.

### Future directions

There are educational efforts in some medical schools in Spain to improve the exposure and understanding of pathology in medical students. These efforts and innovative measures are not only valuable for Spanish medical schools but could potentially be incorporated in the teaching methodology of other countries based on their resources and interests. A recent digital pathology teaching program developed by the University of Valencia faculty has received great satisfaction rates from students and has contributed to a better understanding of the role of the pathologist.^59^

To better teach pathology to students, Spanish medical schools can consider some strategies like incorporating pathology into the medical curriculum from the early years, integrating it with anatomy, physiology, and biochemistry, and reinforcing histological and anatomical correlations in advanced subjects like dermatology. Emphasizing clinical relevance through case studies, patient presentations, and clinical-pathological conferences to illustrate real-world applications of pathological principles is required. Additions of anatomic pathology mentors for students and increasing inter-institutional agreements on a national and international basis are key factors to increase students’ interest in pathology.

We encourage medical schools to provide students with hands-on experience in pathology laboratories, including microscopy, histology, and molecular techniques. Sometimes, hands-on experience in pathology could be limited due to the high volume of daily cases and the rather restricted number of pathologists who can see slides alongside students. That is why simulation-based medical education (SBME), which is a technique used in other specialties such as surgery, can play a significant role in shaping pathology learning in the future. Several studies have compiled information on this topic and highlighted key evaluable characteristics of SBME (team training, feedback, curriculum integration, etc.) so it can be implemented optimally and provide students with assessments of their competence in situations that correlate to actual clinical practice in pathology.^60^

The specific competencies in anatomic pathology for Spain are explored in depth by Dr. Enrique Poblet, who conveyed in his publication that a medical student need not acquire the skills of a pathologist but rather understand when to send a biopsy or additional laboratory tests to this specialty, how reports work, and gain basic knowledge of histopathological manifestations of diseases.^61^ Additionally, the Objective Structured Clinical Examination (OSCE) is emphasized as a reliable, standardized method for assessing clinical performance, ensuring objective evaluation across multiple competencies.^62^

Another frontier is the use of digital pathology platforms to provide students with access to virtual microscopy, digital images, and online resources, enhancing their learning experience and preparing them for the digital age. Virtual slide banks and video lectures on digital slide exploration are excellent tools to expand the possibilities of learning about anatomic pathology. The most recent data on the Spanish medical curriculum expose that only 12.5 % of anatomic pathology subjects include content on digital pathology.^6^ Digital pathology is revolutionizing cancer diagnostics by enabling faster, more accurate diagnoses through digital imaging, artificial intelligence (AI) algorithms, and computer-aided techniques. Automated whole slide imaging (WSI) scanners now produce high-resolution images, integrating imaging into anatomical, clinical, and molecular pathology. Recent US Food and Drug Administration approvals of WSI scanners and AI algorithms, such as for prostate cancer, have paved the way for AI-driven primary diagnoses. These advancements enhance precision medicine and streamline pathology workflows, marking a significant shift in computational histopathology.^63^

Lastly, the invitation of expert pathologists to deliver guest lectures and workshops, providing students with exposure to different subspecialties and cutting-edge techniques might be helpful. Several publications by Alcaraz-Mateos et al. highlight the importance of showcasing techniques performed by pathologists that are beyond the scope of a microscope, such as the fine-needle aspiration (FNA) and grossing techniques. A novel simulator model for FNA and core-needle biopsy cytology training enhances student competency through structured assessment tools like Objective Structured Assessment of Technical Skills (a variant of OSCE) and Debriefing Assessment for Simulation in Healthcare.^64^, ^65^, ^66^ Similarly, the implementation of gross dissection anatomical models in pathology education provides a safe, standardized alternative to traditional methods, improving student understanding of resection margins and macroscopic diagnoses.^67^

## Institutional review board

This project does not involve research on human subjects. It does not include the collection, use, or analysis of identifiable private information or biospecimens. As such, this study is not subject to institutional review board (IRB) review and qualifies as IRB-exempt.

## Author contributions

FRA and MKH were responsible for the concept and design. FRA and SRO were responsible for data collection. MKH was responsible for data analysis. EAM, KM and LC were responsible for the review and addition of supplementary relevant information. All authors contributed to the writing of the manuscript and approved its final version.

## Funding

The article processing fee for this article was funded by an 10.13039/501100023150Open Access Award given by the Society of ‘67, which supports the mission of the Association for Academic Pathology to produce the next generation of outstanding investigators and educational scholars in the field of pathology. This award helps to promote the publication of high-quality original scholarship in *Academic Pathology* by authors at an early stage of academic development.

## Declaration of competing interest

The authors declare that they have no known competing financial interests or personal relationships that could have appeared to influence the work reported in this paper.
